# Supplementary material for: Clonal diversity predicts persistence of SARS-CoV-2 epitope-specific T-cell response
Source: Commun Biol. 2022 Dec 9;5:1351. doi: 10.1038/s42003-022-04250-7 (PMC9734123; doi:10.1038/s42003-022-04250-7)
Supplement: Supplementary file 2 — Supplementary Information [file 42003_2022_4250_MOESM2_ESM.pdf]

List of Supplementary Materials

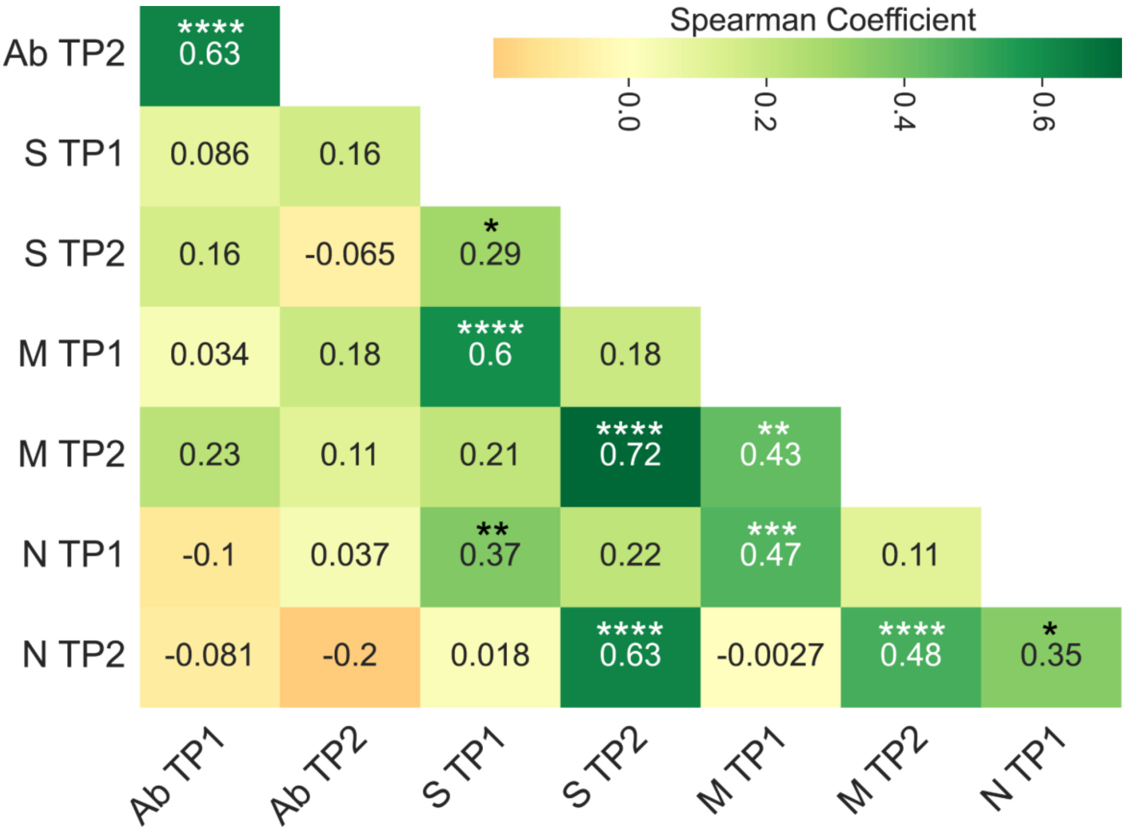

Supplementary Figure 1. Correlation of different parts of immune response. Spearman correlation between humoral (Ab) and cellular responses to different SARS-CoV-2 antigens (S, M, and N proteins).

*$p \leq 0.05$; **$p \leq 0.01$; ***$p \leq 0.001$; ****$p \leq 0.0001$.

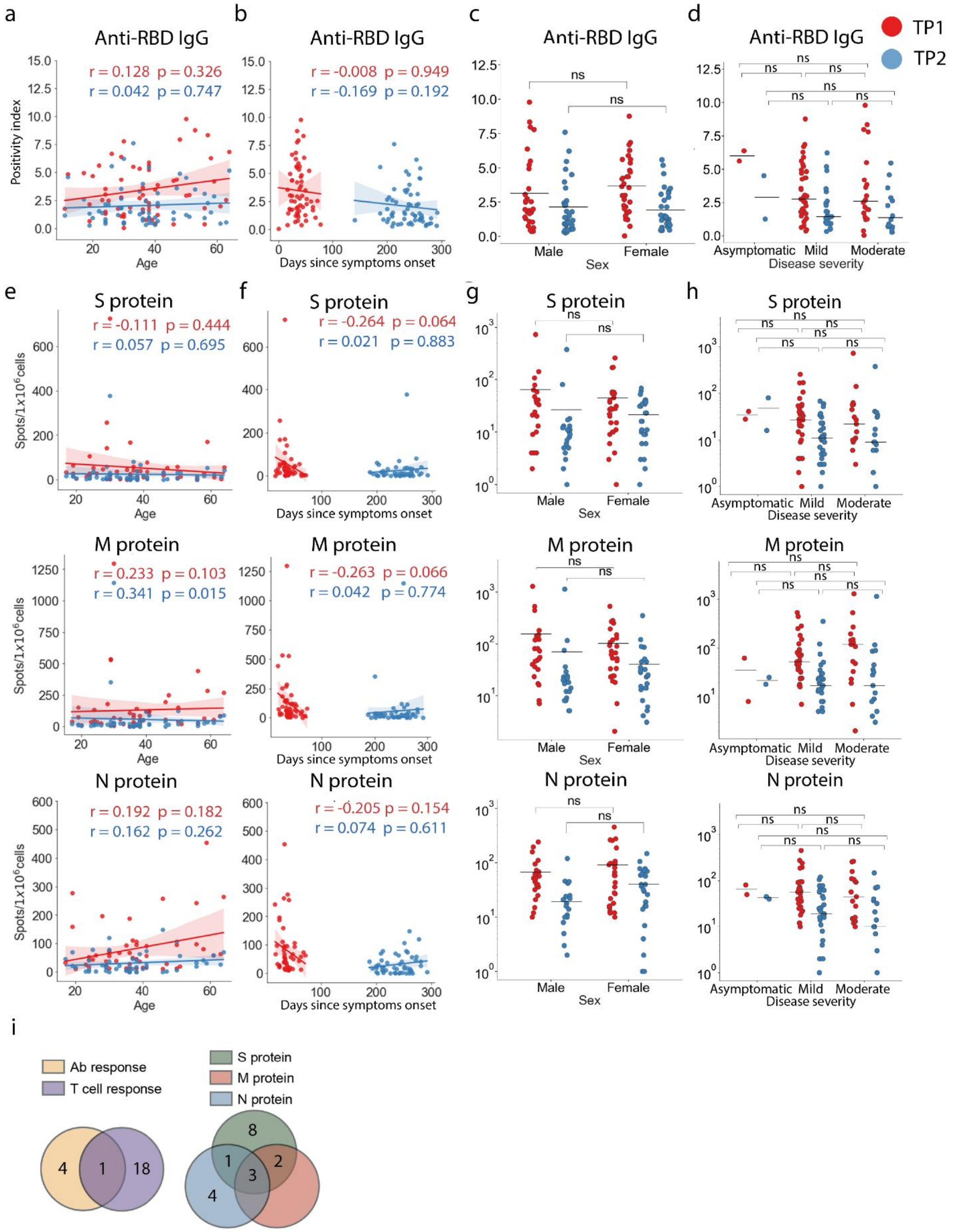

Supplementary Figure 2. Impact of donor age, sex, sampling time-point and disease severity on antibody and T cell response to different SARS-CoV-2 antigens. a Spearman correlation between donor age and antibody titers was measured by ELISA; **b** Spearman correlation between

time after the onset of the disease or positive PCR test and antibody titers; **c** effect of sex on antibody levels; Mann-Whitney test; **d** effect of disease severity on antibody levels; Mann-Whitney test; **e** Spearman correlation between donor age and magnitude of T cell response to S, M, or N protein as measured by IFN-γ ELISpot; **f** Spearman correlation between time after onset of the disease or positive PCR test and magnitude of T cell response to S, M, or N protein; **g** effect of sex on the magnitude of T cell response to S, M, or N protein. r = correlation coefficient; Mann-Whitney test; **h** effect of disease severity on the magnitude of T cell response to S, M, or N protein. r = correlation coefficient; Mann-Whitney test; **i** Venn diagram plotting the intersection of CP with increased level of antibody and T cell response (left) and with increased T cell responses to the S, M and N proteins (right).

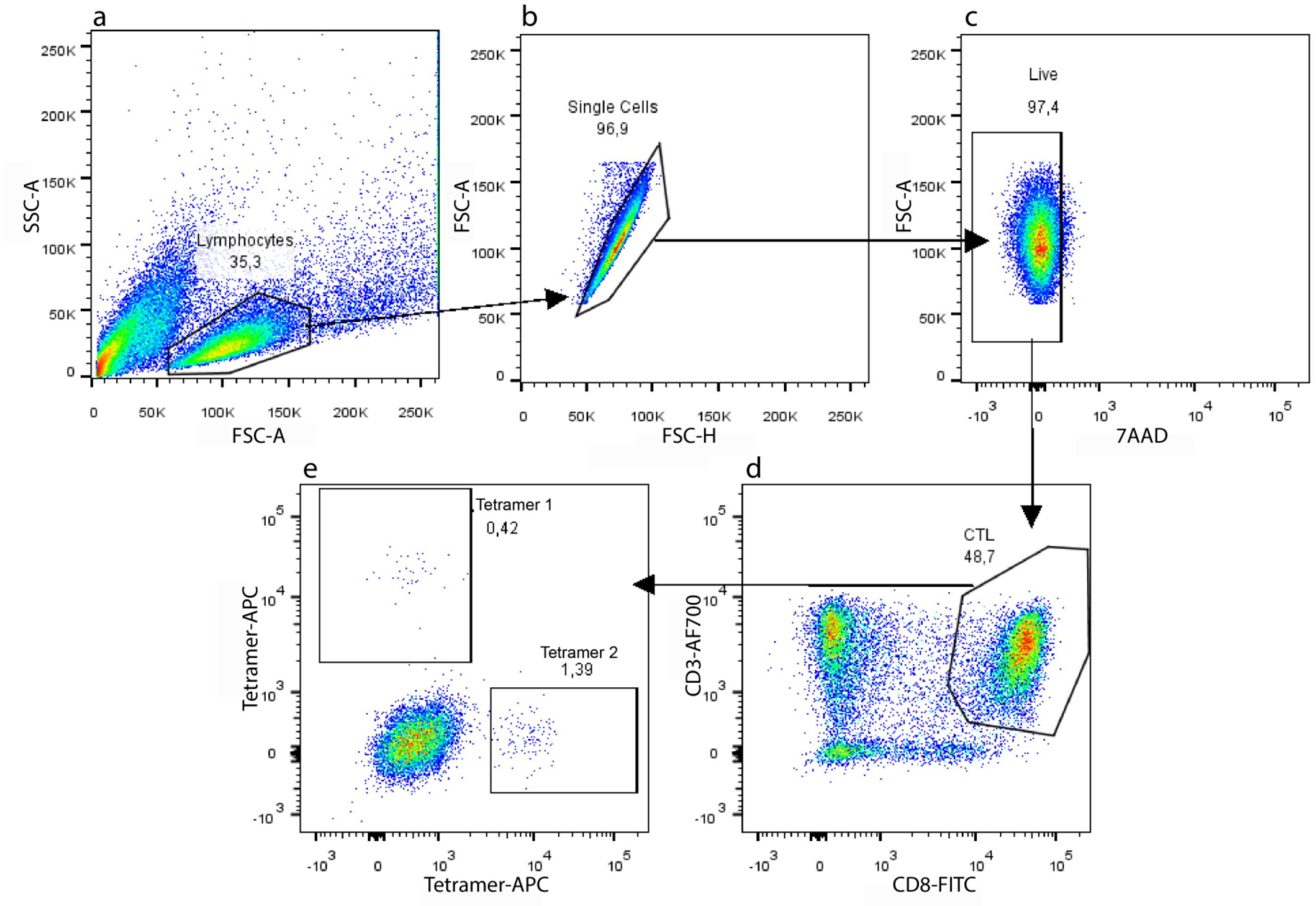

Supplementary Figure 3. Flow cytometry gating strategy. a Total lymphocytes were gated based on forward scatter (FSC-A)/side scatter (SSC-A); **b** singlets were gated based on area and high FSC-A signal; **c** live cells were gated based on FSC-A and absence of 7AAD staining; **d** cytotoxic T cells were gated based on CD3 and CD8 positivity; **e** epitope-specific T cells were gated based on MHC-tetramer-PE or -APC positivity.

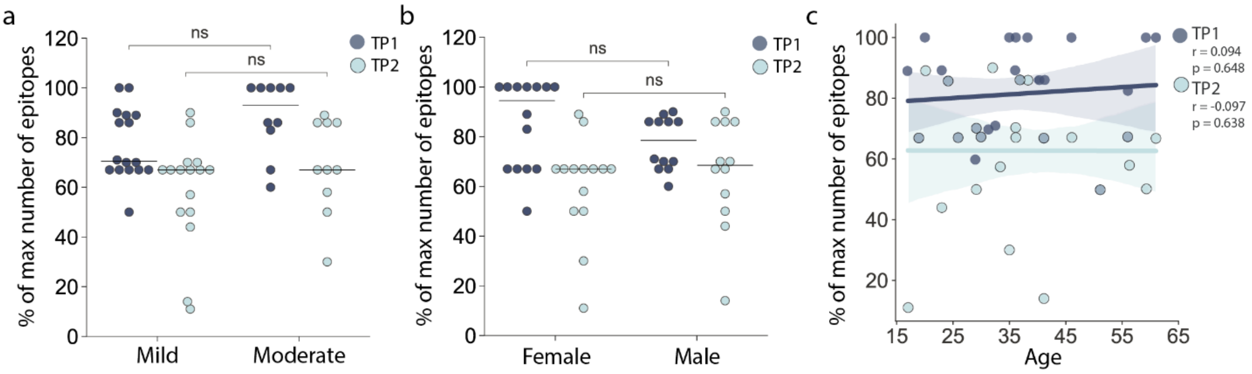

Supplementary Figure 4. Impact of disease severity and donor age and sex on proportion of recognized epitopes. a Effect of disease severity on proportion of recognized epitopes out of the total number of tested epitopes; Mann-Whitney test; **b** effect of sex on proportion of recognized epitopes out of the total number of tested epitopes; Mann-Whitney test; **c** Spearman correlation between donor age and proportion of recognized epitopes out of the total number of tested epitopes. r = correlation coefficient; Mann-Whitney test.

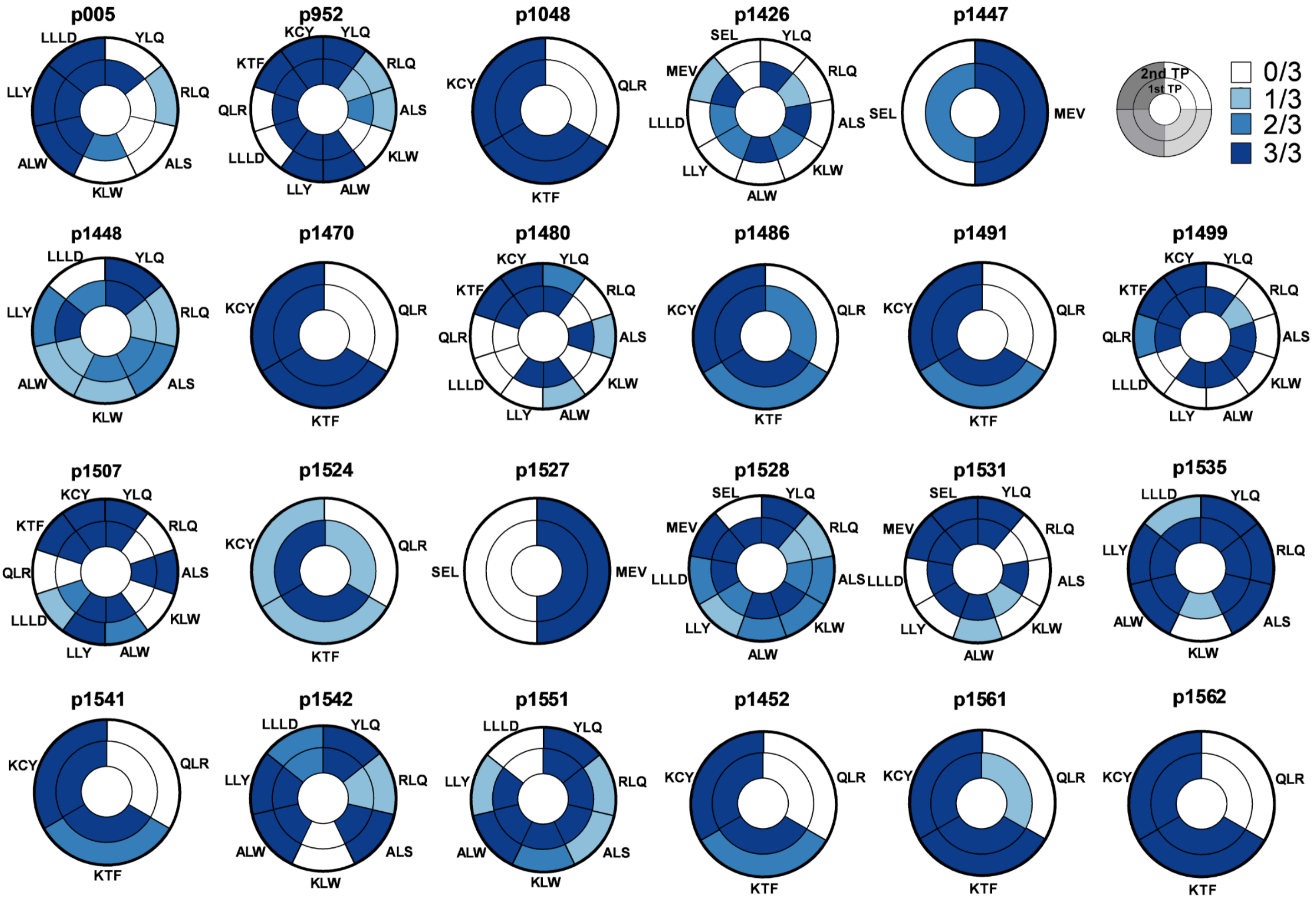

Supplementary Figure 5. Change in frequency of antigen-specific cells after rapid in vitro expansion. Data show relative antigen-specific response at two time-points (TP is inner circle, TP2 is outer circle). Donor ID is indicated on top of each pie chart. Each segment corresponds to one epitope, which is indicated outside by its three- or four-letter code. Color indicates the number of wells with MHC-tetramer^{+} cells.

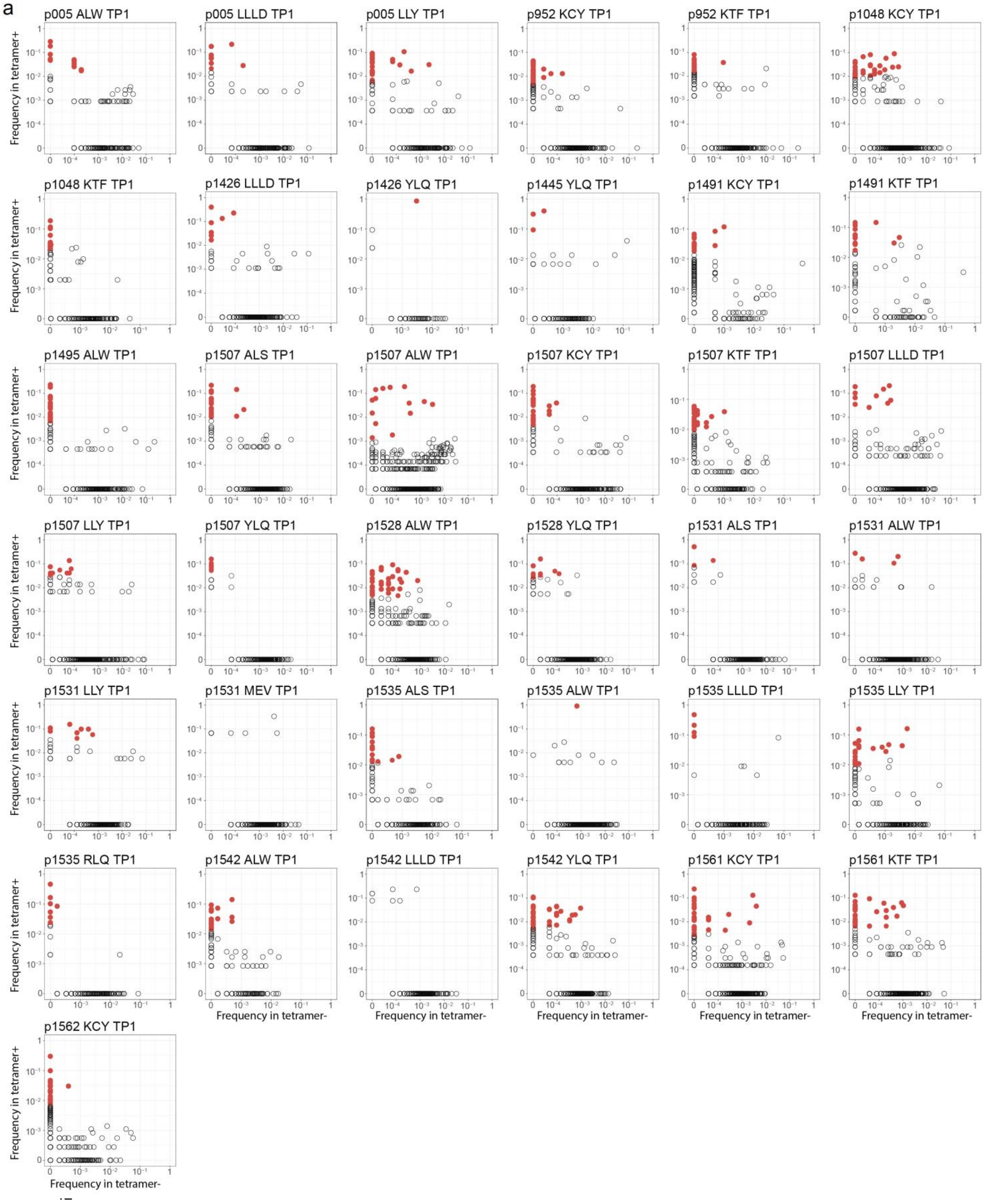
a
p005 ALW TP1
p005 LLLD TP1
p005 LLY TP1
p952 KCY TP1
p952 KTF TP1
p1048 KCY TP1
p1048 KTF TP1
p1426 LLLD TP1
p1426 YLQ TP1
p1445 YLQ TP1
p1491 KCY TP1
p1491 KTF TP1
p1495 ALW TP1
p1507 ALS TP1
p1507 ALW TP1
p1507 KCY TP1
p1507 KTF TP1
p1507 LLLD TP1
p1507 LLY TP1
p1507 YLQ TP1
p1528 ALW TP1
p1528 YLQ TP1
p1531 ALS TP1
p1531 ALW TP1
p1531 LLY TP1
p1531 MEV TP1
p1535 ALS TP1
p1535 ALW TP1
p1535 LLLD TP1
p1535 LLY TP1
p1535 RLQ TP1
p1542 ALW TP1
p1542 LLLD TP1
p1542 YLQ TP1
p1561 KCY TP1
p1561 KTF TP1
p1562 KCY TP1
Frequency in tetramer+
Frequency in tetramer-

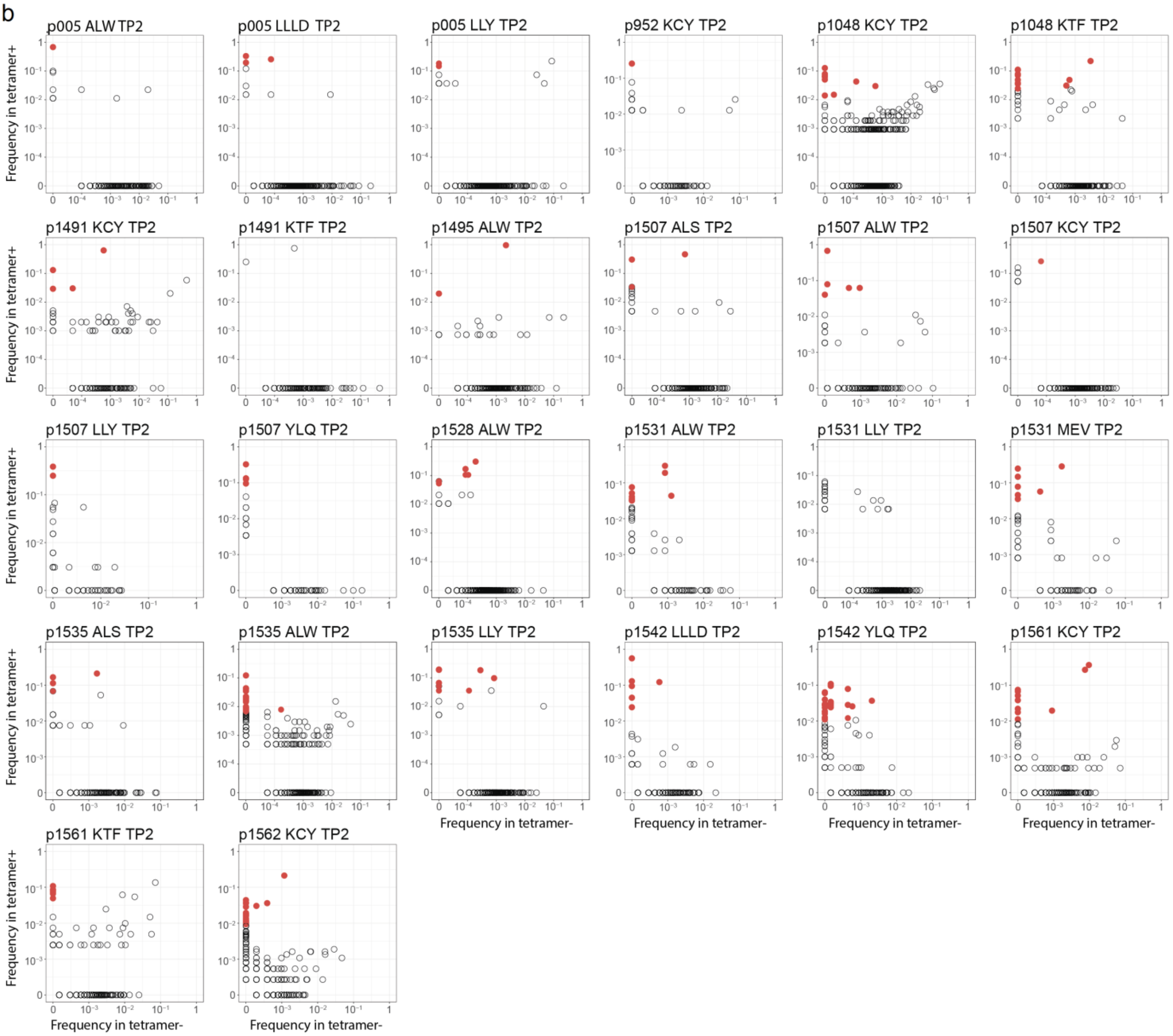

Supplementary Figure 6. Enriched epitope-specific T cell clonotypes. Enrichment plots show frequencies of CDR3β sequences in the MHC-tetramer^{-} and the MHC-tetramer^{+} populations at (**a**) TP1 and (**b**) TP2. Red dots represent clonotypes that are strongly (>10-fold) and significantly ($p < 10^{-12}$, Fisher's exact test) enriched in the MHC-tetramer^{+} population. Donor ID, epitopes and time point are listed at top.

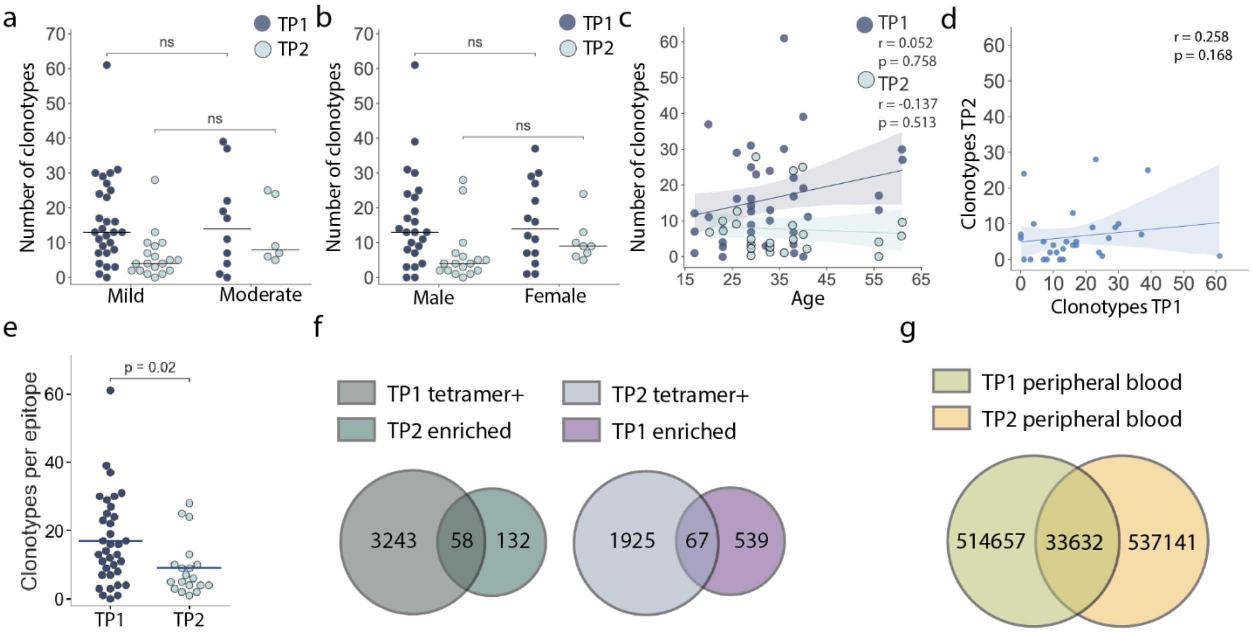

Supplementary Figure 7. Impact of disease severity and donor age and sex on number of clonotypes, correlation between the number of clonotypes at different time-points and intersections of different populations. a Effect of disease severity on number of clonotypes; Mann-Whitney test; **b** effect of sex on number of clonotypes; Mann-Whitney test; **c** Spearman correlation between donor age and number of clonotypes; **d** Spearman correlation between the number of clonotypes at TP1 and TP2. r = correlation coefficient; **e** number of specific clonotypes for epitopes generating a response in three wells. Mann-Whitney test, statistically significant values are annotated; **f**, **g** Venn diagram plotting the intersection of (f) all MHC-tetramer^{+} clones and enriched fractions and (g) peripheral blood clones between the two time-points.

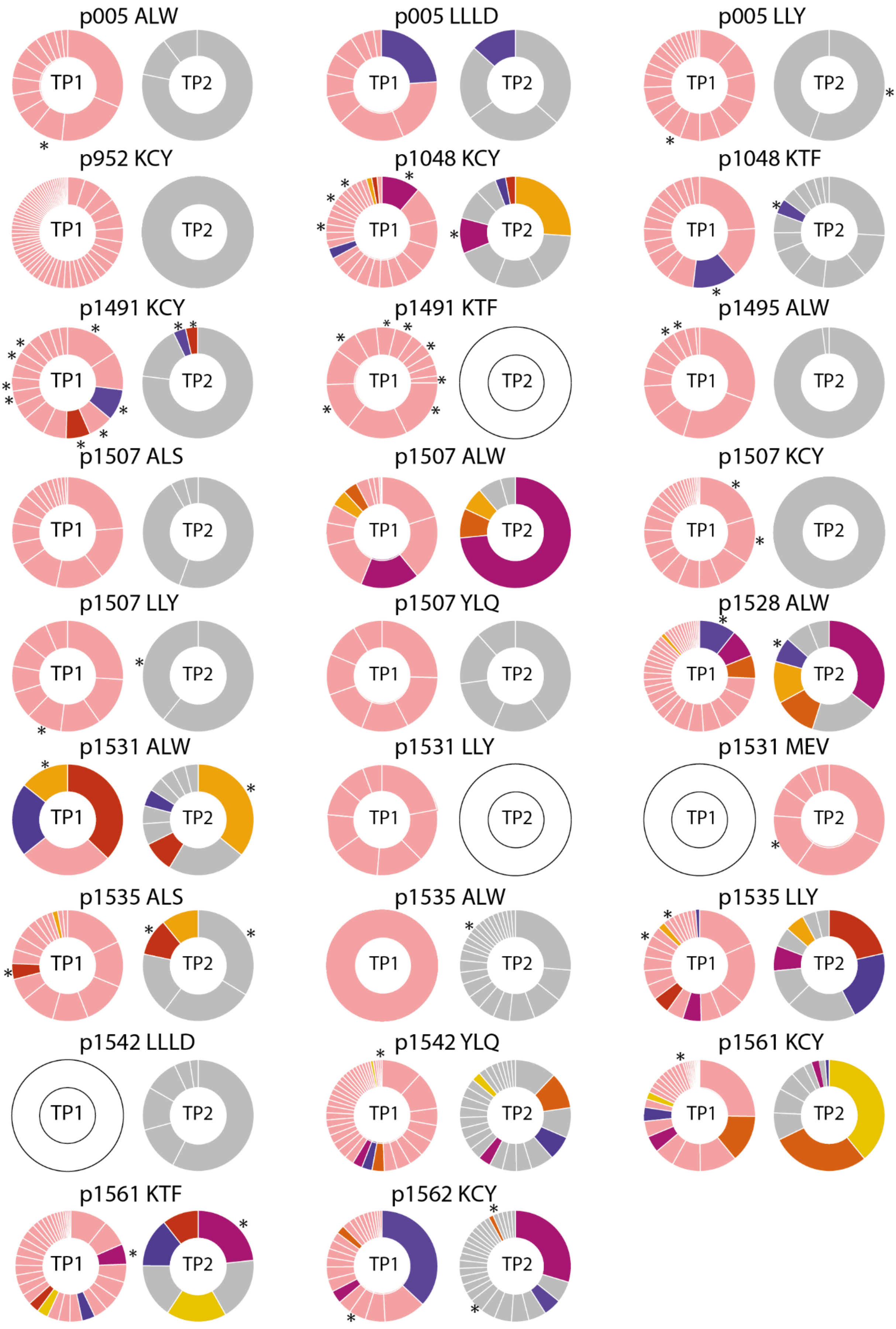

Supplementary Figure 8. Clonal structure of CD8^{+} epitope-specific populations where clones intersecting between the two time-points were found. Pie charts represent the share of the

clonotype in the epitope-specific repertoire at TP1 and TP2. Pink indicates clonotypes found only at TP1, gray indicates clonotypes found only at TP2, other colors indicate clonotypes found at both time-points. Asterisks indicate clonotypes found in total repertoires.

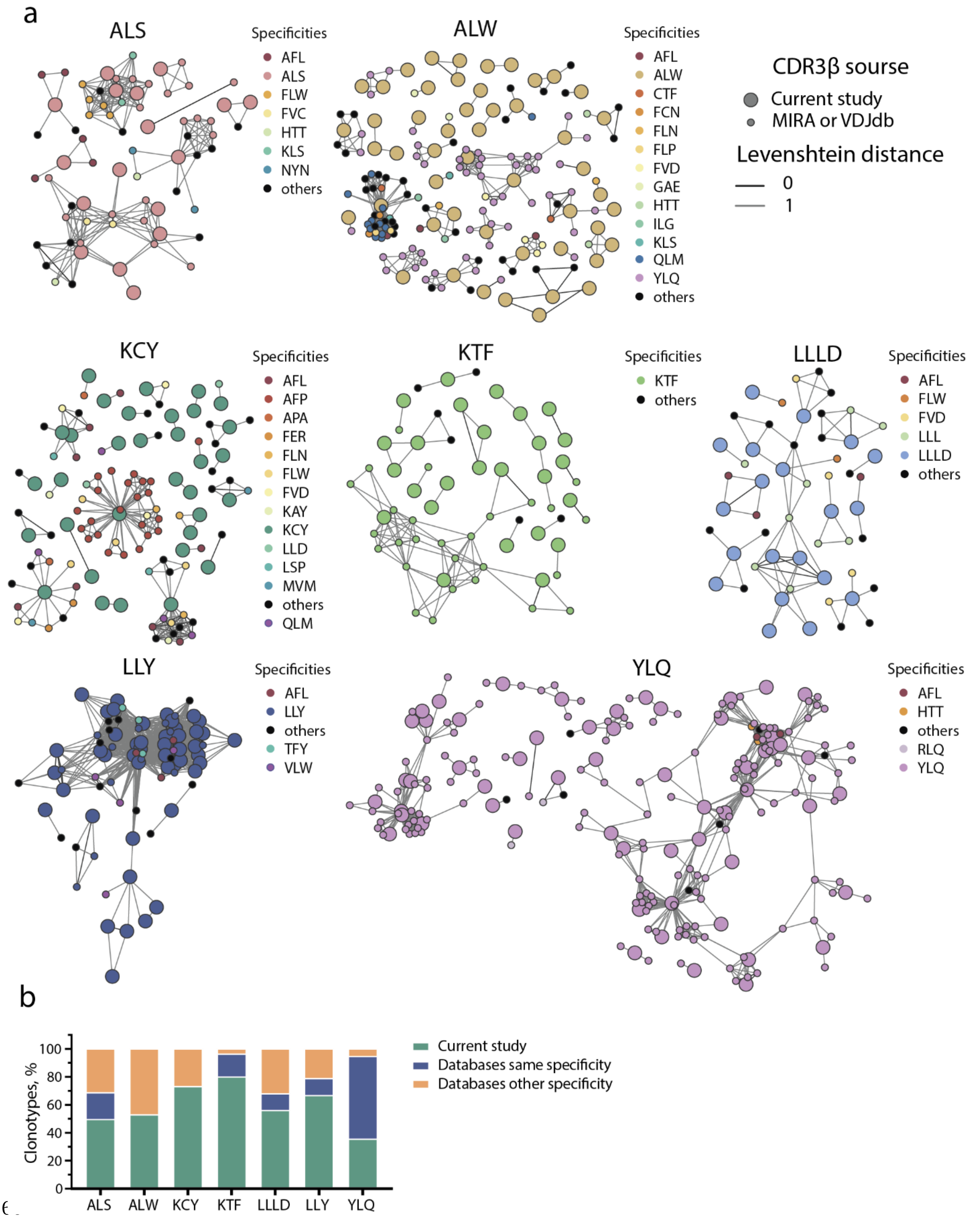

Supplementary Figure 9. SARS-CoV-2 epitope-specific CDR3β amino acid clonotypes form clusters with different levels of similarity. a Nodes representing individual CDR3β sequences. Lines show groups of similar sequences at Levenshtein distance of 1 (grey) or 0 (black). Colors indicate epitope specificities from the current study or from the MIRA and VDJdb databases. Big

circles indicate CDR3 from the current study, small circles are from MIRA or VDJdb. Only clusters with two or more members are shown; **b** fraction of similar sequences (Levenshtein distance ≤ 1) to CDR3 from the current study (yellow) that are also annotated in the MIRA or VDJdb databases with the same (blue) or differing specificity (green).

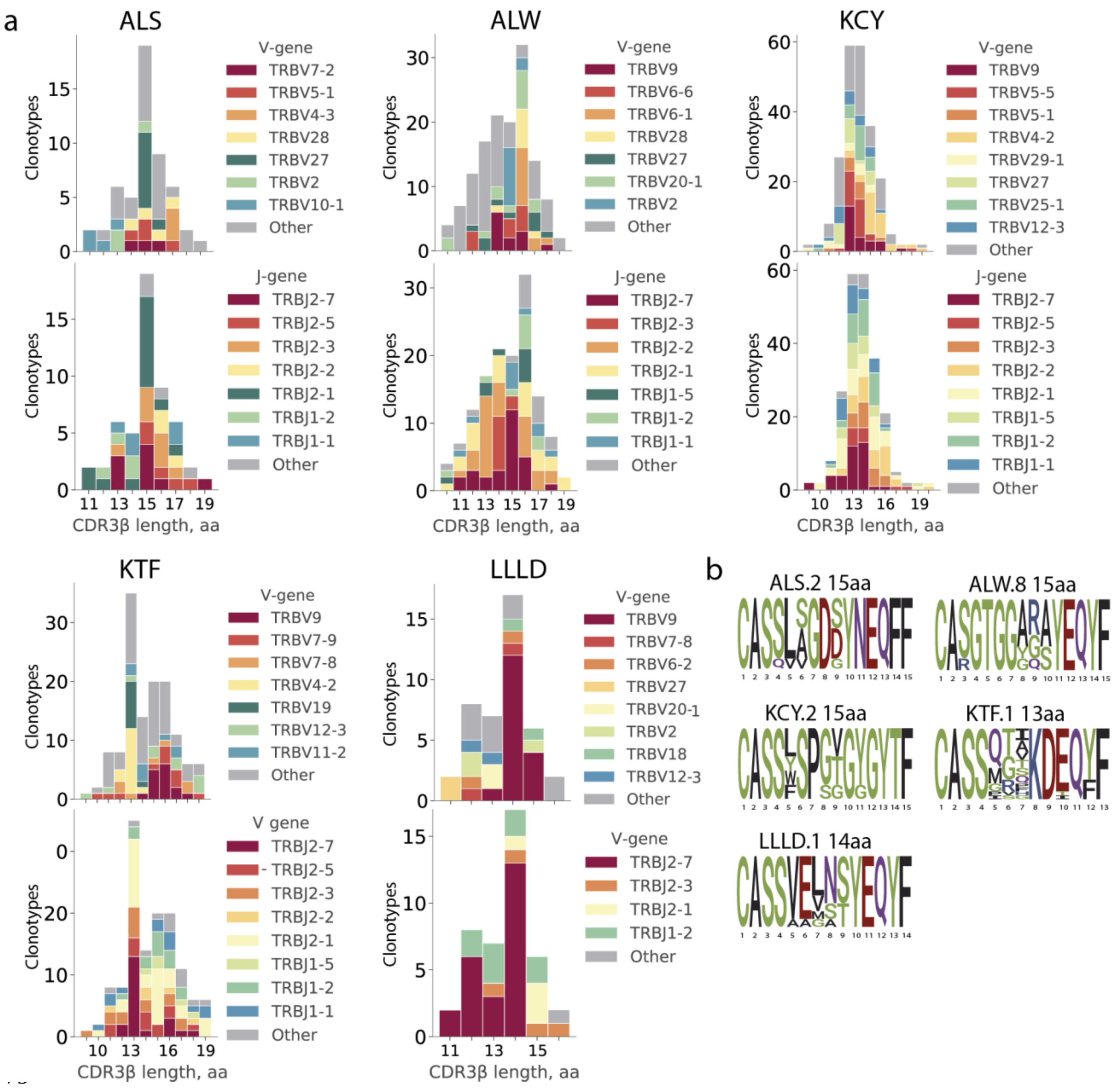

Supplementary Figure 10. V and J gene usage of epitope-specific clonotypes and position-weight matrices for CDR3β sequences. a Histograms of V (upper plots) and J gene usage (lower plots); **b** Position-weight matrices for CDR3β sequences with the most common length found in epitope-specific clusters. Cluster numbers correspond to numbers shown in (Fig. 4a).

Supplementary Table 1. Information about convalescent patients (CD) and healthy donors (HD) recruited in study.

Group	ID	Sex	Age	TP1	TP2	Severity of disease
CP	p1426	M	17	21	209	Mild
CP	p1428	F	47	25	213	Mild
CP	p1436	F	29	25	200	Mild
CP	p1437	M	28	25	251	Mild
CP	p1445	M	32	33	211	Mild
CP	p1446	F	36	25	193	Mild
CP	p1447	F	59	34	204	Mild
CP	p1448	M	37	30	217	Moderate/severe
CP	p1452	F	19	40	208	Mild
CP	p1463	M	38	35	202	Moderate/severe
CP	p1465	M	19	31	210	Moderate/severe
CP	p1466	M	38	38	226	Mild
CP	p1470	F	41	32	200	Mild
CP	p1476	M	30	34	254	Moderate/severe
CP	p1477	F	30	35	220	Moderate/severe
CP	p1480	M	29	37	200	Moderate/severe
CP	p1481	F	30	49	212	Mild
CP	p1482	F	36	39	266	Moderate/severe
CP	p1486	F	46	42	260	Moderate/severe
CP	p1487	M	46	42	260	Mild
CP	p1491	M	56	17	232	Mild
CP	p1495	M	41	45	257	Mild

CP	p1499	F	35	52	263	Moderate/severe
CP	p1507	M	29	39	237	Mild
CP	p1515	F	64	32	235	Moderate/severe
CP	p1521	F	36	64	292	Mild
CP	p1524	F	35	38	235	Moderate/severe
CP	p1526	M	50	41	261	Mild
CP	p1527	F	51	44	264	Mild
CP	p1528	F	20	41	261	Moderate/severe
CP	p1531	M	23	24	247	Mild
CP	p1532	F	56	20	243	Moderate/severe
CP	p1535	F	38	52	281	Moderate/severe
CP	p1537	M	37	50	271	Mild
CP	p1538	F	50	34	251	Moderate/severe
CP	p1541	F	41	41	256	Moderate/severe
CP	p1542	M	40	49	262	Mild
CP	p1543	F	38	39	251	Asymptomatic
CP	p1550	F	58	32	187	Mild
CP	p1551	M	24	35	246	Mild
CP	p1561	F	61	63	272	Mild
CP	p1562	M	30	68	277	Mild
CP	p1565	M	63	72	281	Mild
CP	p1569	F	39	35	240	Moderate/severe
CP	p1576	F	24	34	243	Mild
CP	p005	M	33	34	213	Mild
CP, HD	p1048	F	26	25	187	Asymptomatic

CP, HD	p952	M	36	18	241	Mild
CP, HD	p006	M	24	21	180	Mild
CP, HD	p859	F	25	60	254	Mild
HD	p846	F	24	N/A	N/A	N/A
HD	p1018	F	23	N/A	N/A	N/A
HD	p1032	N/A	N/A	N/A	N/A	N/A
HD	p1187	N/A	N/A	N/A	N/A	N/A
HD	p1305	M	26	N/A	N/A	N/A
HD	p1440	F	30	N/A	N/A	N/A
HD	p818	F	N/A	N/A	N/A	N/A
HD	p1203	F	N/A	N/A	N/A	N/A
HD	p1184	F	N/A	N/A	N/A	N/A
HD	p021	F	N/A	N/A	N/A	N/A
HD	p815	M	N/A	N/A	N/A	N/A
HD	p258	M	N/A	N/A	N/A	N/A
HD	p931	M	N/A	N/A	N/A	N/A
HD	p933	M	N/A	N/A	N/A	N/A
HD	p944	M	N/A	N/A	N/A	N/A

CP - convalescent patient, HD - healthy donor, F - female, M - male, N/A - not available

Supplementary Table 2. HLA typing of CP and HD used for rapid epitope-specific expansions

ID	HLA-A1*	HLA-A1*	HLA-B1*	HLA-B1*	HLA-C1*	HLA-C1*
p1426	02:01:01:01	24:02:01:01	38:01:01:01	40:01:02	03:04:01:01	12:03:01:01
p1445	02:01:01:01	03:01:01:01	44:02:01:01	44:27:01:01	05:01:01:02	07:04:01
p1447	31:01:02:01	31:01:02:01	40:01:02	55:02:01:03	01:02:01:01	03:04:01:01
p1448	02:01:01:01	26:01:01:01	27:05:02:01	39:01:01:05	07:02:01:03	12:03:01:01
p1452	03:01:01:01	29:01:01:01	35:01:01:05	44:02:01:01	04:01:01	16:04:01:01
p1463	01:01:01:01	32:01:01:01	27:05:02:05	37:01:01:01	01:02:01:01	06:02:01:01
p1466	11:01:01:01	32:01:01:01	08:01:01:02	52:01:01:02	07:02:01:01	12:02:02:01
p1470	03:01:01:01	23:01:01:01	35:01:01:05	44:03:01:19	04:01:01	04:09N
p1480	02:01:01:01	03:01:01:03	40:01:02	58:01:01:03	03:02:02:05	03:04:01:01
p1482	01:01:01:01	68:12:01	15:01:01:01	18:03:01:01	06:02:01:01	07:01:01
p1486	01:01:01:01	03:01:01:01	35:01:01:05	52:01:01:02	04:01:01	12:02:02:01
p1491	03:01:01:01	24:02:01:01	35:01:01:05	35:03:01	04:01:01	04:01:01
p1495	02:01:01G	23:01:01G	27:05:02G	44:03:01G	02:02:02G	04:01:01G
p1499	02:01:01G	03:01:01G	07:17	51:01:01G	02:02:02G	07:02:01G
p1507	02:01:01G	03:01:01G	13:02:01G	15:01:01G	03:03:01G	06:02:01G
p1524	03:01:01:01	26:01:01:01	08:01:01:01	51:01:01:01	07:01:01	14:02:01
p1527	25:01:01:01	25:01:01:01	13:02:01:01	40:01:02	03:04:01:01	06:02:01:01
p1528	02:01:01:01	25:01:01:01	18:01:01	40:01:02	01:02:01:01	03:04:01:01
p1531	01:01:01:01	02:01:01:01	15:01:01:01	40:01:02	03:03:01:01	03:04:01:01

p1532	02:01:01:01	03:01:01:01	07:02:01:01	40:01:02	03:04:01:01	07:02:01:03
p1535	02:01:01:01	02:01:01:01	27:05:02:05	27:05:02:10	01:02:01:01	01:02:01:01
p1537	01:01:01:01	24:02:01:01	08:01:01:01	37:01:01:01	06:02:01:01	07:01:01
p1541	03:01:01:01	68:01:01:02	35:03:01	51:01:01:10	04:01:01	05:01:01:02
p1542	02:01:01:01	02:01:01:01	44:02:01:01	49:01:01:01	07:01:01	16:02:01:01
p1550	11:01:01:01	25:01:01:01	15:01:01:01	18:01:01	04:01:01:05	12:03:01:01
p1551	02:01:01:01	68:01:01:02	07:02:01:01	44:02:01:01	05:01:01:02	07:02:01:03
p1561	03:01:01:01	03:01:01:01	07:02:01:01	07:02:01:01	07:02:01:03	07:02:01:03
p1562	01:01:01:01	03:01:01:01	07:02:01:01	13:02:01:01	06:02:01:01	07:02:01:03
p1569	02:01:01:01	11:01:01:01	35:08:01:01	57:01:01:01	04:01:01:28	06:02:01:01
p005	1:01	2:01	8:01:01	44	N/A	N/A
p1048	01:01:01:01	03:01:01:01	41:02	52:01:01:02	07:01:01	17:01
p952	2:01	03:01	49	51	4	7
p859	2:01	2:01	7:02	7:02	7:02	7:02
p1018	02:01:01:01	1:01	35:01:00	27:05:00	1:02	4:01
p818	2:01	24:02:00	40:01:00	49:01:00	3:04	7:01
p1203	02:01:01:01	03:01:01:01	40:01:01	40:02:01	2:02:02	3:04:01
p1184	02:01:01:01	03:01:01:01	13:02:01	57:01:01	6:02	6:02

86

Supplementary Table 3. Proportion of epitope-specific clonotypes from MHC - tetramer-positive clonotypes.

ID	Epitope	TP1 epitope-specific clonotypes/ TP1 tetramer-positive clonotypes	TP2 epitope-specific clonotypes/ TP2 tetramer-positive clonotypes
p005	ALW	13/71 (18.3%)	3/11 (27.3%)
p005	LLLD	10/31 (32.3%)	4/9 (44.4%)
p005	LLY	24/64 (37.5%)	2/13 (15.4%)
p1048	KCY	29/85 (34.1%)	9/279 (3.2%)
p1048	KTF	16/47 (34%)	13/30 (43.3%)
p1426	ALS	12/126 (9.5%)	-
p1426	LLLD	7/35 (20%)	-
p1426	YLQ	1/4 (25%)	-
p1445	YLQ	2/23 (8.7%)	-
p1491	KCY	17/152 (11.2%)	4/53 (7.5%)
p1491	KTF	13/64 (20.3%)	0/2 (0%)
p1495	ALW	10/96 (10.4%)	2/22 (9.1%)
p1507	ALS	14/93 (15%)	4/21 (19%)
p1507	ALW	14/325 (4%)	5/24 (20.8%)
p1507	KCY	25/66 (37.9%)	1/11 (9.1%)
p1507	KTF	31/172 (18%)	-
p1507	LLLD	12/97 (12.4%)	-
p1507	LLY	9/44 (20.5%)	2/28 (7.1%)
p1507	YLQ	7/31 (22.6%)	4/30 (13.3%)
p1528	ALW	37/258 (14.4%)	7/16 (43.8%)
p1528	YLQ	11/51 (21.6%)	-
p1531	ALS	3/10 (30%)	-
p1531	ALW	4/23 (17.4%)	10/50 (20%)
p1531	LLY	8/37 (21.6%)	0/38 (0%)
p1531	MEV	0/11 (0%)	7/42 (16.7%)
p1535	ALS	16/67 (23.9%)	5/44 (11.4%)
p1535	ALW	1/12 (8.3%)	23/582 (4%)
p1535	LLLD	4/9 (44.4%)	-
p1535	LLY	22/82 (26.8%)	9/16 (56.3%)
p1535	RLQ	6/15 (40%)	-
p1542	ALW	19/108 (17.6%)	-
p1542	LLLD	0/7 (0%)	6/34 (17.6%)
p1542	YLQ	38/99 (38.4%)	25/69 (36.2%)
p1561	KCY	30/137 (21.9%)	10/73 (13.7%)
p1561	KTF	27/81 (33.3%)	6/82 (7.3%)
p1562	KCY	23/234 (9.8%)	28/484 (5.8%)
p952	KCY	61/161 (37.9%)	1/45 (2.2%)
p952	KTF	30/108 (27.8%)	-
