# Supplementary material for: Clonal diversity predicts persistence of SARS-CoV-2 epitope-specific T-cell response
Source: Commun Biol. 2022 Dec 9;5:1351. doi: 10.1038/s42003-022-04250-7 (PMC9734123; doi:10.1038/s42003-022-04250-7)
Supplement: Supplementary file 3 — Description of Additional Supplementary Files [file 42003_2022_4250_MOESM3_ESM.pdf]

Description of Additional Supplementary Files

File name: Supplementary Data 1
Description: The source data behind the graphs in the paper.

File name: Supplementary Data 2
Description: List of epitope-specific CDR3β sequences.
