# Supplementary material for: Clonal diversity predicts persistence of SARS-CoV-2 epitope-specific T-cell response
Source: Commun Biol. 2022 Dec 9;5:1351. doi: 10.1038/s42003-022-04250-7 (PMC9734123; doi:10.1038/s42003-022-04250-7)
Supplement: Supplementary file 6 — Reporting Summary [file 42003_2022_4250_MOESM6_ESM.pdf]

nature portfolio

Corresponding author(s): Grigory A. Efimov

Last updated by author(s): Oct 31, 2022

Reporting Summary

Nature Portfolio wishes to improve the reproducibility of the work that we publish. This form provides structure for consistency and transparency in reporting. For further information on Nature Portfolio policies, see our Editorial Policies and the Editorial Policy Checklist.

Statistics

For all statistical analyses, confirm that the following items are present in the figure legend, table legend, main text, or Methods section.

n/a	Confirmed	
☐	☒	The exact sample size (*n*) for each experimental group/condition, given as a discrete number and unit of measurement
☐	☒	A statement on whether measurements were taken from distinct samples or whether the same sample was measured repeatedly
☐	☒	The statistical test(s) used AND whether they are one- or two-sided<br>*Only common tests should be described solely by name; describe more complex techniques in the Methods section.*
☐	☒	A description of all covariates tested
☐	☒	A description of any assumptions or corrections, such as tests of normality and adjustment for multiple comparisons
☐	☒	A full description of the statistical parameters including central tendency (e.g. means) or other basic estimates (e.g. regression coefficient) AND variation (e.g. standard deviation) or associated estimates of uncertainty (e.g. confidence intervals)
☐	☒	For null hypothesis testing, the test statistic (e.g. *F*, *t*, *r*) with confidence intervals, effect sizes, degrees of freedom and *P* value noted<br>*Give P values as exact values whenever suitable.*
☒	☐	For Bayesian analysis, information on the choice of priors and Markov chain Monte Carlo settings
☒	☐	For hierarchical and complex designs, identification of the appropriate level for tests and full reporting of outcomes
☒	☐	Estimates of effect sizes (e.g. Cohen's *d*, Pearson's *r*), indicating how they were calculated

Our web collection on statistics for biologists contains articles on many of the points above.

Software and code

Policy information about availability of computer code

Data collection	For TCR sequences matching open source databases VDJdb (https://vdjdb.cdr3.net/) and ImmuneCODE (https://www.adaptivebiotech.com/immunecode/) were used.
Data analysis	TCR repertoire data were analyzed using MIXCR (https://github.com/milaboratory/mixcr), MIGEC (https://github.com/mikessh/migec), and VDJtools software (https://github.com/mikessh/vdjtools) with default settings. Epitope-specific TCR sequences were matched against VDJdb and ImmuneCODE datasets using the VDJmatch tool (https://github.com/antigenomics/vdjmatch). Graphs were plotted using “igraph” R package version 1.2.6. TCR logos were plotted using “ggseqlogo” package version 0.1.<br>R markdown notebooks and Python file are used for data analysis are available at https://github.com/LabTransplantImmunology/tcr-seq-pipline/

For manuscripts utilizing custom algorithms or software that are central to the research but not yet described in published literature, software must be made available to editors and reviewers. We strongly encourage code deposition in a community repository (e.g. GitHub). See the Nature Portfolio guidelines for submitting code & software for further information.

Data

Policy information about availability of data

All manuscripts must include a data availability statement. This statement should provide the following information, where applicable:

- Accession codes, unique identifiers, or web links for publicly available datasets
- A description of any restrictions on data availability
- For clinical datasets or third party data, please ensure that the statement adheres to our policy

All data is available, including accession codes and web links for publicly available datasets.

Human research participants

Policy information about studies involving human research participants and Sex and Gender in Research.

Reporting on sex and gender	All findings may be applied to all sexes and genders. Sex was determined based on self-reporting. This information was allowed to be shared according to informed consent signed by the participants.
Population characteristics	Covariate-relevant characteristics: concomitant diseases and COVID-19 treatment strategies.
Recruitment	Participants volunteered for participation in this study. Only participants with COVID-19 confirmed by positive SARS-CoV-2 RT-PCR test were recruited.
Ethics oversight	National Medical Research Center for Hematology ethical committee (N 150, 02.07.2020)

Note that full information on the approval of the study protocol must also be provided in the manuscript.

Field-specific reporting

Please select the one below that is the best fit for your research. If you are not sure, read the appropriate sections before making your selection.

☒ Life sciences ☐ Behavioural & social sciences ☐ Ecological, evolutionary & environmental sciences

For a reference copy of the document with all sections, see nature.com/documents/nr-reporting-summary-flat.pdf

Life sciences study design

All studies must disclose on these points even when the disclosure is negative.

Sample size	No sample-size calculation was performed. Sample size was limited due to small number of COVID-19 convalescent individuals at the time of sample collection (march-may 2020).
Data exclusions	No data was excluded.
Replication	All attempts at replication of T cell response and antibody level were successful. Sequencing resulted were not replicated due to lack of material.
Randomization	Participants were allocated into experimental groups based on presence or absence of positive SARS-CoV-2 RT-PCR test.
Blinding	Blinding was not relevant to the study because we studied specific group of convalescent individuals.

Reporting for specific materials, systems and methods

We require information from authors about some types of materials, experimental systems and methods used in many studies. Here, indicate whether each material, system or method listed is relevant to your study. If you are not sure if a list item applies to your research, read the appropriate section before selecting a response.

Materials & experimental systems

n/a	Involved in the study
☐	☒ Antibodies
☒	☐ Eukaryotic cell lines
☒	☐ Palaeontology and archaeology
☒	☐ Animals and other organisms
☒	☐ Clinical data
☒	☐ Dual use research of concern

Methods

n/a	Involved in the study
☒	☐ ChIP-seq
☐	☒ Flow cytometry
☒	☐ MRI-based neuroimaging

Antibodies

Antibodies used

CD3-AF700 (Mouse IgG2a, κ, Clone OKT3, Sony, Cat.No.2186700)
CD8-FITC (Mouse IgG1, κ, Clone RPA-T8, Sony, Cat.No.2105250)

Validation

CD3-AF700 (according to manufacturer's website each lot of this antibody is quality control tested by immunofluorescent staining with flow cytometric analysis).
CD8-FITC (according to manufacturer's website each lot of this antibody is quality control tested by immunofluorescent staining with flow cytometric analysis).

Flow Cytometry

Plots

Confirm that:

☒ The axis labels state the marker and fluorochrome used (e.g. CD4-FITC).

☒ The axis scales are clearly visible. Include numbers along axes only for bottom left plot of group (a 'group' is an analysis of identical markers).

☒ All plots are contour plots with outliers or pseudocolor plots.

☒ A numerical value for number of cells or percentage (with statistics) is provided.

Methodology

Sample preparation

30 mL of venous blood from donors was collected into EDTA tubes (Sarstedt) and subjected to Ficoll (Paneco) density gradient centrifugation (400 x g, 30 min). Isolated PBMCs were washed with PBS containing 2 mM EDTA and used for assays or frozen in fetal bovine serum containing 7% DMSO. For rapid in vitro expansion, we used PBMCs of donors. Cells were split between three wells and incubated for 10–12 days in RPMI 1640 culture medium supplemented with 10% normal human A/B serum, 1 mM sodium pyruvate, 25 ng/mL IL-7, 40 ng/mL IL-15, and 50 ng/mL IL-2 (Miltenyi) at a final volume of 2 ml/well. Half of the medium was replaced on days 3, 5, and 7. A mix of patient HLA-specific peptides of SARS-CoV-2 in DMSO or MES buffer / isopropanol mixture was added on day 0. The final concentration of each peptide in the medium was 10 ng/mL. Antigen-specific cells were detected by staining (with CD3-AF700, CD8-FITC, 7AAD, and with combinations of two different peptide-MHC-tetramer complexes conjugated with streptavidin-allophycocyanin and streptavidin-R-phycoerythrin.

Instrument

Aria III cell sorter (BD Biosciences)

Software

FlowJo Software (version 10.6.1)

Cell population abundance

Abundance of tetramer-positive fraction was in range 1000 to 20000 cells. Abundance of tetramer-negative fraction was in range 100000 to 500000 cells. Purity of samples was determined using post-sort FACS analysis.

Gating strategy

Total lymphocytes were gated based on forward scatter (FSC)/side scatter (SSC-A). Singlets were gated based on area and high FSC-A signal. Live cells were gated based on FSC-A and absence of 7AAD staining. Cytotoxic T cells were gated based on CD3 and CD8 positivity. Epitope-specific T cells were gated based on MHC-tetramer-PE or -APC positivity. Boundaries between 'positive' and 'negative' populations were defined with FMO-staining controls and lay between 10^3 and 10^4.

☒ Tick this box to confirm that a figure exemplifying the gating strategy is provided in the Supplementary Information.
